# Nanovesicles From *Lactobacillus johnsonii* N6.2 Reduce Apoptosis in Human Beta Cells by Promoting AHR Translocation and IL10 Secretion

**DOI:** 10.3389/fimmu.2022.899413

**Published:** 2022-06-09

**Authors:** Leandro D. Teixeira, Natalie A. Harrison, Danilo R. da Silva, Clayton E. Mathews, Claudio F. Gonzalez, Graciela L. Lorca

**Affiliations:** ^1^ Department of Microbiology and Cell Science, Genetics Institute, Institute of Food and Agricultural Sciences, University of Florida, Gainesville, FL, United States; ^2^ Department of Pathology, Immunology, and Laboratory Medicine, College of Medicine, University of Florida, Gainesville, FL, United States

**Keywords:** AHR, *lactobacillus johnsonii* N6.2, human beta cell line βlox5, human monocyte cell line THP-1, human pancreatic islets, IL10, nanovesicles, STAT3

## Abstract

*L. johnsonii* N6.2 releases nano-sized vesicles (NVs) with distinct protein and lipid contents. We hypothesized that these NVs play a central role in the delivery of bioactive molecules that may act as mechanistic effectors in immune modulation. In this report, we observed that addition of NVs to the human pancreatic cell line βlox5 reduced cytokine-induced apoptosis. Through RNAseq analyses, increased expression of *CYP1A1, CYP1B1, AHRR*, and *TIPARP* genes in the aryl hydrocarbon receptor (AHR) pathways were found to be significantly induced in presence of NVs. AHR nuclear translocation was confirmed by confocal microscopy. The role of NVs on beta cell function was further evaluated using primary human pancreatic islets. It was found that NVs significantly increased insulin secretion in presence of high glucose concentrations. These increases positively correlated with increased *GLUT6* and *SREBF1 mRNA* and coincided with reduced oxidative stress markers. Furthermore, incubation of NVs with THP-1 macrophages promoted the M2 tolerogenic phenotype through STAT3 activation, expression of AHR-dependent genes and secretion of IL10. Altogether, our findings indicate that bacterial NVs have the potential to modulate glucose homeostasis in the host by directly affecting insulin secretion by islets and through the induction of a tolerogenic immune phenotype.

## Introduction

Type 1 diabetes is caused by an autoimmune response against the beta cells in pancreatic islets, characterized by infiltration of islet-specific autoreactive CD8^+^ T cells, macrophages and B cells, resulting in destruction of the islets. While genetic profile is the main determinant of type 1 diabetes risk, a variety of environmental triggers have been associated with beta cell damage, such as viral infections and metabolic/oxidative stress [reviewed in (1)]. Associations between microbiota dysbiosis, intestinal permeability and abnormal mucosal immune activation have been proposed as environmental determinants of type 1 diabetes onset. While preclinical data supports this hypothesis (2), analyses of longitudinal data from The Environmental Determinants of Diabetes in the Young (TEDDY) failed to identify a pro-inflammatory microbiota (3). Interestingly, TEDDY data also reported an association between decreased risk of islet autoimmunity and early supplementation of probiotics (between the age of 0-27 days) while the responses were highly variable in other age groups (4). Probiotics are “live microorganisms which, when administered in adequate amounts, confer a health benefit on the host” (5). Many microorganisms are commonly used in dietary supplements as probiotics such as *Lactobacillus* and *Bifidobacterium* species, which are considered Generally Regarded as Safe (GRAS) microbes. The mechanistic action of probiotics are diverse and strain specific (6). We hypothesize that the variability observed on the TEDDY cohort may be due to the use of a diverse array of probiotic products not specifically selected for type 1 diabetes prevention.

Our preclinical studies showed that the gut microbiota of BioBreeding Diabetes Resistant (BB-DR) rats was significantly different from Diabetes Prone (BB-DP) rats, where a higher prevalence of *Lactobacillus and Bifidobacterium* species were observed in BB-DR animals (7). Supporting the role of the microbiota as modulators of type 1 diabetes onset, we showed that oral administration of *Lactobacillus johnsonii* N6.2 (herein referred to as *L. johnsonii*), isolated from BB-DR animals, decreased type 1 diabetes onset in BB-DP rats when compared to the administration of *L. reuteri* (8). We also found that diabetes prevention correlated with a T_H_17 cell bias with elevated IL-23 levels within the mesenteric lymph nodes (9). Further *in vitro* studies indicated that the modification of dendritic cells (DCs) by oral feeding of *L. johnsonii* contributed to the T_H_17 bias.

Further mechanistic studies showed that *L. johnsonii* produces a metabolite that strongly inhibits the enzyme Indoleamine 2,3-dioxygenase (IDO) activity *in vitro* as well its mRNA levels *in vivo*. Free tryptophan is the substrate for IDO to produce kynurenine. It is expressed by DCs, which is a key regulator of T cell development and immune responses. An *in vivo L. johnsonii* feeding assay performed in BB-DP rats showed *L. johnsonii* induced lower levels of intestinal IDO gene transcription, which correlated with decreased concentrations of kynurenine in blood plasma (10). It was also found that administration of *L. johnsonii* to BB-DP rats reduced inflammasome activation through decreased expression of nod-like receptor protein 3(NLRP3) and decreased maturation of Caspase 1 in the ileum (11). Taken together, these findings strongly suggest that *L. johnsonii* can inhibit the inflammasome assembly, reducing levels of the pro-inflammatory cytokine IL-1β, which would have direct consequences on the activation of pro-inflammatory T cell phenotypes. These results are in agreement with reports that indicate a deficiency in NLRP3 can prevent type 1 diabetes in NOD mice (12).

In the immunophenotyping analysis of PBMCs from healthy subjects in a human clinical trial, the administration of *L. johnsonii* resulted in a progressive increase in the frequencies of monocytes and NK cells, however, neither B cell nor DC frequency showed differences between the groups during treatment. Interestingly, the increase in monocyte and NK cells co-occurred with a decrease in the percentage of CD4^+^ cells (13). This observed decrease in CD4^+^ cells did not correlate with changes in the percentages of Teff, however, the activation status of Th1 cells transiently increased in the probiotic group, an expected result for probiotics with immune stimulatory effects (14). A similar trend was observed with the Treg subset in the *L. johnsonii* treatment group after the washout period. The findings in healthy humans treated with *L. johnsonii* strongly support the hypothesis that *L. johnsonii* modulates the immune response, possibly through induction of a tolerogenic Th17 phenotype, as well as through inhibition of inflammatory pathways. Taken together, our results indicate that *L. johnsonii* is able to modulate host responses locally and systemically.

One of the limitations of our preclinical and clinical studies has been the absence of identified mechanistic effectors of *L. johnsonii* activity. As a step in that direction, we have recently characterized extracellular vesicles (herein referred as nanovesicles, NVs) secreted by *L. johnsonii* (15). Similar to mammalian cells, both Gram-negative and Gram-positive bacteria are able to release NVs (16). These NVs are evolutionarily conserved, have a spherical shape composed of proteolipid bilayers, and carry specific subsets of lipids, nucleic acids, proteins and metabolites (17). Proteomic and lipidomic analysis showed that NVs secreted by *L. johnsonii* have a unique protein and lipid composition that is distinct from the *L. johnsonii* cell membrane (15). NV surface-exposed proteins were evaluated for their potential to generate an immune response in human hosts. Plasma from individuals administered *L. johnsonii* contained IgA and IgG antibodies against NV and T285_RS00825 (named Sdp) protein domains *in vivo*. Altogether, these results demonstrate that *L. johnsonii* NVs have the potential to mediate host interactions through immune modulation.

Based on these findings, we hypothesize that NVs secreted by *L. johnsonii* can mediate local and systemic responses either by direct targeting of specific cell types or systemically through immune modulation. Here, we show that NVs from *L. johnsonii* can modulate apoptosis in human beta cells, promote insulin and IL-10 secretion by human islets and induce the polarization of THP-1 macrophages towards a M2b phenotype.

## Materials and Methods

### Growth and Cell Fractionation of *L. johnsonii*

*Lactobacillus johnsonii* was grown in de Man, Rogosa, Sharpe (MRS) media as previously described (8). NVs and membrane isolation were performed from *L. johnsonii* cultures grown in exosome-free MRS media (15). Isolated NVs were immediately quantified using Nanosight NS300 (Malvern instruments Ltd, Malvern, UK) and aliquots were stored at -80°C until use.

### Culture and Treatment of Human Cell Lines

The pancreatic cell line βlox5 was obtained from Dr. Fred Levine (Sanford Children’s Health Research Center, La Jolla, CA) (18). Caco-2, Jurkat, and THP-1 cell lines were obtained from ATCC (Gaithersburg, MD, USA). βlox5 cells were cultured in Dulbecco’s Modified Eagle Medium (DMEM) medium supplemented with 10% heat inactivated FBS (Sigma-Aldrich, Saint Louis, MO), 1% penicillin and streptomycin solution containing 10,000 units of penicillin and 10 mg of streptomycin/ml (Sigma-Aldrich, Saint Louis, MO), 0.2% of BSA, HEPES (15 mM), pH=7, and 5 ml of MEM non-essential amino acids (Sigma-Aldrich, Saint Louis, MO). Caco-2 cells were cultured in Eagle’s minimum essential medium (EMEM) supplemented with 15% heat inactivated FBS and 2% of penicillin and streptomycin solution. Cells were cultured in RPMI 1640 medium supplemented with 10,000 units of penicillin, 10 mg of streptomycin, and 10% or 20% heat inactivated FBS (for Jurkat or THP-1 cells, respectively). Where indicated, cytokines were added at TNFα (1,000 U/mL), IFNγ (750 U/mL) and IL-1β (75 U/mL). For the THP-1/βlox5 co-cultures, THP-1 cells were seeded in the inserts at 5 x 10^5^ cells/well and activated by adding phorbol 12-myristate-13-acetate (PMA) at 100 nM, for 48 h, at 37°C. Media was then removed, and inserts were transferred to a six well plate containing 6 x 10^5^ βlox5 cells. Vehicle control (10 µL of media) or NVs at 10^10^ particles/mL were added to the top chamber containing the THP-1 cells at 37°C. After 5 h, cells from both chambers were harvested and used for qRT-PCR and Western blot analysis.

### Co-Culture of Cell Lines

Monocyte THP-1 cells were co-cultured with βlox5 cells using 4 µm pore size cell culture inserts (Thermo Scientific, Waltham, MA). THP-1 cells were seeded in the inserts at 5 x 10^5^ cells/well and activated by adding phorbol 12-myristate-13-acetate (PMA) at 100 nM, for 48 h, at 37°C in 5% CO_2_ incubator. Media was then removed and inserts were transferred to a 6 well plate containing 6 x 10^5^ βlox5 cells. Vehicle control (10 µl of complete media) or NV at 10^10^ particles/ml were added to the top chamber containing the THP-1 cells at 37°C in 5% CO_2_ incubator. After 5 h, cells from both chambers were harvested and individually used for qRT-PCR and Western blot analysis.

### mRNA Isolation and qRT-PCR Analysis

RNA was isolated from cell lines and human pancreatic islets *via* Trizol. Briefly, 300 µl of Trizol (Zymo Research, Irvine, CA) was added to each well. After homogenizing, the Trizol reagent was transferred to an Eppendorf tube containing 300 ul of 95% ethanol and thoroughly mixed. The mixture was transferred to a spin column (Zymo Research, Irvine, CA) and RNA isolation was performed following the manufacturer’s protocol. DNA was removed by treatment with DNase (QIAGEN, Germantown, MD) according the manufacturer’s protocol. RNA quality was monitored on 1% agarose gels, and RNA quantification was performed using Thermo Scientific Nanodrop One Microvolume UV-vis spectrophotometer (Thermo Fisher Scientific, Grand Island, NY). qRT-PCR was performed as described (19). Primer sequences used to determine relative transcript abundance are listed in **Table S1**.

### βlox5 Global Transcriptomic Analysis

After extraction of total RNA, library construction and sequencing were performed by Novogene, (Novogene Co., Davis, CA, USA). 1 µg of RNA from each sample was used for the library preparation. Sequencing libraries were generated using NEBNext^®^ Ultra TM RNA Library Prep Kit for Illumina^®^ (NEB, USA) following manufacturer’s recommendations. mRNA was purified from total RNA using poly-T oligo-attached magnetic beads. Fragmentation was carried out using divalent cations under elevated temperature in NEBNext First Strand Synthesis Reaction Buffer (5X). First strand cDNA was synthesized using random hexamer primers and M-MuLV Reverse Transcriptase (RNase H-). Second strand cDNA synthesis was subsequently performed using DNA Polymerase I and RNase H. In order to select cDNA fragments of preferentially 150~200 bp in length, the library fragments were purified with AMPure XP system (Beckman Coulter, Beverly, USA). Then 3 µl USER Enzyme (NEB, USA) was used with size-selected, adaptor ligated cDNA at 37°C, for 15 min, followed by 5 min at 95°C before PCR. PCR was performed with Phusion High-Fidelity DNA polymerase (NEB, USA), Universal PCR primers and Index (X) Primer. PCR products were purified (AMPure XP system) and library quality was assessed on the Agilent Bioanalyzer 2100 system. The clustering of index-coded samples was performed on a cBot Cluster Generation System using PE Cluster Kit cBot-HS (Illumina) according to the manufacturer’s instructions. After cluster generation, the library preparations were sequenced on an Illumina platform and paired-end reads were generated. Raw data (raw reads) of FASTQ format were firstly processed through fastp (20) to generate clean data (clean reads). Paired-end clean reads were aligned to the reference genome using the Spliced Transcripts Alignment to a Reference (STAR) software. Novogene Co., Ltd Quantification FeatureCounts (21) was used to count the read numbers mapped for each gene, and the RPKM of each gene was calculated based on the length of the gene and read counts mapped to this gene. Differential expression analysis between two conditions/groups was performed using DESeq2 R package (17). The resulting *p* values were adjusted using the Benjamini-Hochberg method for controlling the False Discovery Rate (FDR). Genes with an adjusted *p* value < 0.05 found by DESeq2 were assigned as differentially expressed. GO term enrichment analysis was used to analyze the biological significance of the differently expressed genes (DEGs) and graphs were generated by R package “GOplot” (22). One-way ANOVA was used to identify genes with significant differences between all groups. Tukey honestly significant difference (HSD) test was used to determine the significance between two groups.

### Apoptosis Assay

Approximately 1x10^6^ βlox5 cells were incubated with NVs at a concentration of 10^8^ or 10^10^ particles (1:100 or 1:10000 cells to nanovesicles, respectively), and cytokines [TNFα (1 U/ml), IFNγ (1 U/ml), and IL-1β (0.056 U/ml)], followed by incubation at 37°C, under 5% CO_2,_ for 6 h. Then, cells attached and in the supernatant were combined by centrifugation at 700 x g for 5 min and washed once with 2 ml of cold βlox5 complete media. Cells were labeled according to the specifications of the BD Annexin V FITC Assay using the BD FACSVerse™ System Kit. Cells were counted using the Accuri C6 flow cytometer (BD Biosciences, Franklin Lakes, NJ, USA). The analysis was performed using FCS Express Version 7 software (DeNovo software, Pasadena, CA, USA), and the gating strategy is shown in **Supplementary Figure 3**.

### Glucose-Stimulated Insulin Secretion From Human Pancreatic Islets

Human pancreatic islets were purchased from ProdoLabs (Aliso Viejo, CA). Islets were cultured in CMRL 1066 medium, supplemented with 10% heat inactivated FBS (Sigma-Aldrich, Saint Louis, MO), 5,000 units of penicillin and 5 mg of streptomycin (Sigma-Aldrich, Saint Louis, MO) at 37°C, in 5% CO2 incubator. After a transport recovery period of 48 h, islets were incubated in starvation media containing a 50:50 mixture of DMEM (no glucose) and Ham’s media, 7.1 mM sodium bicarbonate, 750 µM calcium chloride dehydrated, 2.5% BSA, 1% penicillin/streptomycin solution and 3 mM D-glucose overnight at 37°C, under 5% CO_2_. After 12 h, the starvation media was replaced by KREBB’s media containing either 0.5, 3, 10, or 15 mM D-glucose, and incubated at 37°C, under 5% CO_2_. Samples from the supernatant were collected after 1, 2, and 3 h of incubation, and insulin concentration was determined by ELISA (Mercodia, Winston Salem, NC). The insulin concentration obtained was normalized to the DNA concentration of the islets. DNA was extracted using DNeasy blood and tissue kit (QIAGEN, Germantown, MD) according to the manufacturer’s protocol. Insulin secretion was expressed as mU Insulin/L/ng of DNA. For qRT-PCR analysis, 250 islets equivalent (IEQ) were cultured with 6 x 10^9^ NVs for 5 h in CMRL 1066 culturing medium, supplemented with 10% heat inactivated FBS, 1% penicillin and streptomycin solution (Sigma-Aldrich, Saint Louis, MO) at 37°C in 5% CO2 incubator. RNA was extracted as described above. Each experiment was conducted in triplicates with islets purified from two non-diabetic donors. Demographics are provided in **Table S2**. Details of batch glucose-stimulated insulin secretion (GSIS) are provided in the Supplementary Materials section. Each experiment was conducted in triplicate with islets purified from donors without a diagnosis of diabetes.

### Western Blot Assays

Western blot assays were used to test the presence of STAT3, P-S727-STAT3 and P-Y705-STAT3. β actin quantification was used as the loading control. All antibodies were purchased from Abcam (Cambridge, MA, USA). Total proteins were extracted from βlox5 cells using Radio Immunoprecipitation Assay Buffer (RIPA) containing 150 mM NaCl, 50 mM Tris (pH 8), 1% Triton X-100, and 0.1% sodium dodecyl sulfate (SDS), with Halt™ protease inhibitor cocktail (Thermo Fisher, Waltham, MA, USA). The cell homogenates were centrifuged at 12,000 *g* for 10 min, at 4°C and the protein concentration was measured using Pierce™ BCA Protein Assay Kit (Thermo Fisher Scientific, Waltham, USA). Fifteen micrograms of proteins were separated using 12.5% (v/v) sodium dodecyl sulfate-polyacrylamide gel electrophoresis (SDS-PAGE) and transferred to a polyvinylidene difluoride (PVDF) membrane (Bio Rad, Hercules, CA, USA) using a semi dry transfer system. The membranes were blocked with 5% non-fat milk in 0.1% Tween 20, in saline Tris buffer pH=7.4 (TBS-T) for 1 h at RT. PVDF membranes were incubated with anti-STAT3 and anti-phospho S727 STAT3 at 1/1,000 dilution factor, anti-phospho Y705 STAT3 at 1/500 dilution factor, and anti-actin at 1/10,000 primary antibodies (Abcam, Cambridge, MA, USA) over night at 4°C. Membranes were washed with tris buffered saline containing 1% tween 20 (TBS-T), three times, for 5 minutes at RT after blocking and incubation with primary antibodies. Subsequently, the membranes were incubated with horseradish peroxidase-conjugated secondary antibody at 1/10,000 for 1 h at RT and washed three times with TBS-T. The enhanced chemoluminescence method (ProSignal^®^ ECL Reagent; Genesee Scientific, San Diego, CA, USA) was used for visualization *via* the automatic imager FluorChem R (Proteinsimple, San Jose, CA, USA). The relative intensity of the bands visualized in the membranes were quantified with ImageJ software (Free Java software provided by the National Institutes of Health, Bethesda, Maryland, USA). In all cases, β-actin band intensity was used to normalize the bands of each sample. All experiments were done in biological triplicate.

### AHR Nuclear Translocation Assays Using Confocal Microscopy

To visualize changes in the cellular localization of AHR, 3x10^5^ βlox5 cells were cultured on coverslips previously coated with poly D-lysine (Sigma-Aldrich, Saint Louis, MO) for 3 h. AHR translocation assays were initiated by addition of NVs at 10^10^ particles/ml or 2,3,7,8-tetrachlorodibenzo-p-dioxin (TCDD) at 100 nM for 0 and 45 minutes, at 37°C, under 5% CO_2_. Following incubation, the cells were washed three times with ice-cold PBS (pH 7.4), fixed with 4% paraformaldehyde for 10 min, and washed three times with ice cold PBS. Cells were permeabilized with PBS solution containing 0.1% Triton X-100 for 15 min, at 25°C and rinsed three times with PBS, 5 minutes each wash. Cells were incubated with rabbit anti-AHR antibody tagged with R-phycoerythrin (PE)-texas red (Abcam, Cambridge, MA) for 1h at 25°C and subsequently washed three times with PBS. Fluorescence was acquired using a confocal fluorescence microscope (Carl Zeiss, Berlin, Germany) and 3D reconstructions generated by acquiring Z-stacks of 18 images.

### ELISA

Supernatants from cell/islet cultures were used to determine protein levels of IL10, IL1β, TNFα (BD biosciences, Franklin Lakes, NJ, USA) and insulin (Mercodia, Winston Salem, NC, USA) according to the manufacturer’s protocols.

### Statistical Analysis

R studio (RStudio Team, Boston, MA) as well as GraphPad Prism 5.01 software (GraphPad Software, La Jolla, CA, United States) were used for data analysis and visualization. Statistical tests were performed using one-way analysis of variance (ANOVA) to evaluate the effects of treatments on cell lines or human islets. Significance of model terms and treatment comparisons were considered significant at a level of α=0.05, and degrees of freedom were adjusted using the Kenward–Rogers correction. Finally, *post hoc* multiple comparisons were performed with least significant differences.

### Data and Resource Availability

The RNA sequencing data generated in this study are deposited in the NCBI Sequence Read Archive (SRA) under BioProject PRJNA804404.

## Results

### NVs From *L. johnsonii* Reduced the Relative Number of Apoptotic βlox5 Cells

**Figure 1:**
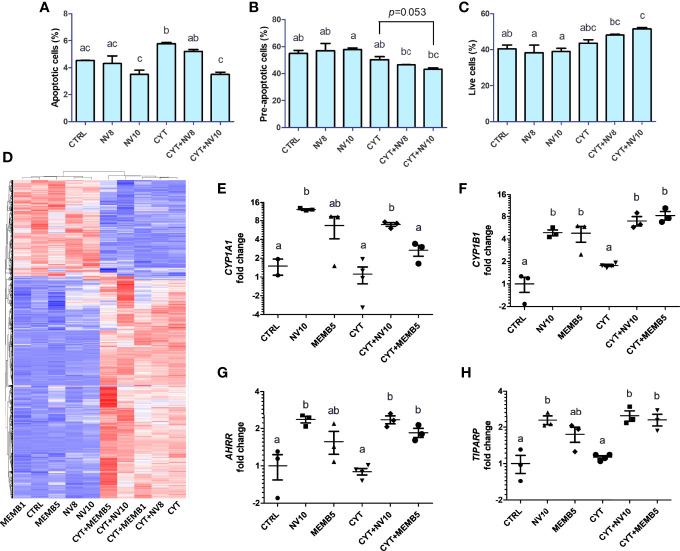
*L. johnsonii*-derived NVs modulate apoptosis rates **(A–C)** and mRNA profiles **(D–H)** in βlox5 cells. The percentage of apoptotic **(A)**, pre-apoptotic **(B)** and live **(C)** cells were determined *via* flow cytometry. Cells were primed with NVs (except on vehicle control and cytokine groups) for 2 h prior to adding the pool of cytokines (IL-1β, TNFα and IFNγ) and further incubation for 6 h. **(D–H)** Total RNA sequencing was performed in βlox5 cells incubated in presence or absence of NVs or MEMB. Cells were primed with NVs or MEMB (except on vehicle control and cytokines groups) for 2 h prior to adding the pool of cytokines (IL-1β, TNFα and IFNγ), and further incubation for 6 h. **(D)** Heatmap depicting hierarchical clustering of the samples. The fold change in expression of *CYP1A1, CYP1B1, AHRR* and *TIPARP* are shown in **(E)**, **(F)**, **(G)** and **(H)**, respectively. Letters on top of each group represent statistical analyses, where different letters denote significant difference (p < 0.05). NV8 and NV10 denote NVs from *L. johnsonii* at 10^8^ and 10^10^ particles/ml, respectively; MEMB1 and MEMB5 denote cell membrane isolates from *L. johnsonii* at 1 and 5 ug/ml, respectively; CYT denotes the pool of cytokines (IL-1β, TNFα and IFNγ); CYT+NV8 and CYT+NV10 denotes cells treated with NVs and the pool of cytokines; CYT+MEMB1 and CYT+MEMB5 denote cells treated with the pool of cytokines and *L. johnsonii* cell membrane isolates at 1 and 5 ug/ml, respectively. Data are expressed as mean ± SD. Letters on top of each bar represent statistical significance (p < 0.05), where bars sharing at least one letter are not significantly different.

Before exhibiting its typical clinical symptoms, type 1 diabetes follows a slow-progressing autoimmune process, comprised of a cascade of immunological steps, culminating with apoptosis of beta cells. It has been shown that secretion of IL-1β, TNFα and IFNγ by local antigen presenting cells (APCs) and infiltrating T cells, are responsible for induction of transcription factors and apoptosis-related proteins, such as NF-κB, caspase-3, p38 and c-Jun N-terminal kinase (JNK) (23). To investigate the effect of *L. johnsonii* on inflammation-induced apoptosis, human βlox5 cells were incubated in presence or absence of NVs at 10^8^ or 10^10^ particles/ml (NV8 or NV10, respectively) for 2 h, at 37°C. Addition of 10^8^ or 10^10^ NV/mL did impact cell viability compared to the vehicle control (4.3%, 3.5%, and 4.5%, respectively). The addition of cytokines significantly (*p*<0.05) increased apoptosis of βlox5 cells. Addition of NV with cytokines prevented apoptosis (**Figures 1A, B**). Similarly, a reduction in pre-apoptotic cells was observed (*p*=0.053). The reduction in apoptotic cells with NV10 also resulted in an increase in live cells, when compared to the control group (**Figure 1C**). In contrast, the addition of NV8 did not affect the number of apoptotic, pre-apoptotic or live cells, suggesting the ability for NVs to protects against apoptosis in the pancreatic beta cell line is dose dependent (**Figures 1A–C**).

In human islets, apoptosis may be triggered by intrinsic and extrinsic pathways, as well as through perforin/granzyme pathway (24). To evaluate the effect of NVs on apoptosis pathways, expression of the pro-apoptotic genes BAX, BIM, FADD and the anti-apoptotic genes BCL-2 and BCL-XL were quantified in βlox5 cells following the ame treatment protocol as immediately above. Expression of these genes was not significantly modified by the addition of NVs or the pool of cytokines (data not shown).

### NVs From *L. johnsonii* Affect βlox5 Gene Expression Levels in a Dose-Dependent Manner

To identify the mechanism by which NVs reduce apoptosis in βlox5 cells, we performed RNAseq analysis of βlox5 cells treated with NVs from *L. johnsonii* at 10^8^ and 10^10^ particles/ml (NV8 and NV10, respectively), in presence or absence of the pool of cytokines. We have recently reported that different proteins and lipids are enriched in NVs when compared to the membranes (MEMB) isolated from *L. johnsonii* (15). To compare the differential host response to NVs or MEMB components, βlox5 cells were challenged with 1 or 5 µg/mL MEMB (MEMB1 and MEMB5, respectively) in the presence or absence of the cytokine combination. MEMB1 and MEMB5 are equivalent to NV concentrations NV8 and NV10, respectively.

The gene expression profile was mostly driven by the addition of cytokines (**Figure 1D**). All differentially expressed genes were classified into functional categories using gene ontology enrichment analyses (GO terms) (**Supplementary Figures 1A–D**).

In absence of an inflammatory environment, the addition of NV8, as well as MEMB1, did not result in significant changes in gene expression. Treatments with NV10 or MEMB5 did not repress the expression of any gene. Interestingly, 21 genes were up regulated by addition of NV10, while MEMB5 up regulated the expression of two genes (**Table 1**). The expression of Cytochrome P450 Family 1 Subfamily B Member 1 (*CYP1B1*) was the only significantly upregulated transcript that overlapped between the N10 and MRMB5. The GO categories enriched by the addition of NV10 were defense response to virus and cellular response to type 1 interferons (**Table 1**; **Supplementary Figure 1**).

**Table 1:** Genes significantly (adjusted *p* value < 0.05) modified by the treatment of βlox5 with *L. johnsonii*-derived NVs or cell membrane isolates when compared to vehicle control.

NV10 vs CTRL			MEMB5 vs CTRL		
Gene name	Fold change(log2)	Adjusted *p* value	Gene name	Fold change(log2)	Adjusted *p* value
*CYP1B1*	2.3	3.37E-12	*CYP1B1*	2.3	3.27E-05
*VIPR1*	1.7	5.71E-11	*TXNIP*	1.2	0.00337
*MX1*	2.4	7.84E-06			
*MX2*	2.8	9.37E-06			
*IFI44L*	3.3	3.02E-05			
*CYP1A1*	3.6	0.000338			
*TIPARP*	1.2	0.000403			
*CDK15*	1.6	0.000979			
*DDX60*	1.0	0.001631			
*EPSTI1*	1.5	0.001781			
*STC2*	1.1	0.00224			
*OAS2*	3.0	0.00224			
*IFI44*	1.3	0.003432			
*SECTM1*	1.3	0.007822			
*AHRR*	1.2	0.007904			
*IL1B*	1.1	0.011816			
*OASL*	1.5	0.012745			
*IFIT1*	1.3	0.024639			
*ALDH1A3*	0.9	0.024639			
*OAS1*	2.1	0.037023			
*IL6*	1.3	0.039443			

Addition of the pool of cytokines induced a strong shift in the expression profile of βlox5 cells. It was found that 1110 genes were significantly upregulated while 419 were downregulated. As expected, GO pathway analysis indicated that most of the upregulated genes are classified in categories related to the regulation of cytokine production and inflammatory responses (**Supplementary Figure 1B**; **Supplementary Table 3**). Under these inflammatory conditions, co-treatment with NV10 (CYT+NV10) was found to upregulate the expression of an additional 119 genes and downregulate the expression of 20 genes. Interestingly, many of the upregulated genes belong to the initial response to viral infection categories, similar to the results observed in absence of the pool of cytokines (**Figure 1A**; **Table 1**). In contrast, the addition of the cytokine pool to MEM5 treated cells resulted in the enrichment of different GO term categories, where upregulated genes belonged to categories related to regulation of vascular endothelial growth, response to toxic substances, and regulation of hormone levels, while the downregulated genes belong to the I band, supramolecular complex/polymer and fiber categories (**Supplementary Figure 1**). The differential effect observed on βlox5 cells following treatment with different cellular components of *L. johnsonii*, indicate that NVs are able to generate a specialized host response when compared to cell membranes from the same bacterium.

### *L. johnsonii*- Derived NVs Suggest a Link Between AHR Activation and RNA Sensing in βlox5 Cells

In absence of inflammatory conditions, the addition of NV10 significantly induced the 2’,5’-oligoadenylate synthetase (OAS) and the AHR pathways (**Table 1**). The OAS pathway is an IFN-stimulated antiviral response activated by viral or bacterial RNA (25). In humans, the OAS family is composed of three enzymatically active enzymes, OAS1, OAS2 and OAS3, all of which were significantly induced in presence of NV10, but not by NV8, MEMB1, or MEMB5. As expected, all three OAS genes were significantly induced upon addition of the pool of cytokines (where INFγ is a component). Interestingly, the expression of those genes was further induced by the addition of NV10 from *L. johnsonii*, with the exception of OAS2 (**Table 1**). Similar results were observed on the enzymatically inactive OASL, which is induced by RIG-1 and MAVS oligomerization, and is crucial in the stabilization of the OAS complex (**Table 1**). Taken together, these findings suggest that nucleic acids present in NVs are sensed by the βlox5 cells. Other genes involved in the sensing and response to nucleic acids, such as *IFI44L, MX1, MX2* and *DDX60*, followed a similar pattern of stimulation in presence of NV10 (**Table 1**).

Genes in the AHR pathway were among the most upregulated genes induced by NV10. AHR is a ubiquitous transcription factor that is activated in response to environmental and metabolic cues. In the inactive form, AHR is located on the cytoplasm, associated in a multiprotein complex that includes the chaperone heat-shock protein 90 (HSP90), the co-chaperone p23, and the hepatitis B virus X-associated protein (XAP2). Upon binding to a ligand, conformational changes in AHR result in its translocation into the nucleus, where it changes interaction partners by binding to the AHR nuclear translocator (ARNT). The AHR/ARNT complex binds to specific sequences in the promoter thereby controlling the expression of several genes (26). Transcription of cytochrome P450 (CYP1) family members, including subfamily A member 1 (*CYP1A1*) and subfamily B member 1 (*CYP1B1*), is regulated by AHR activation. Our data shows that 10^10^ NV particles/ml from *L. johnsonii* significantly increased the expression of *CYP1A1* and *CYP1B1*. The addition of MEM5 significantly increased the expression of *CYP1B1* and *AHRR* (*p*<0.05), while the changes induced in *CYP1A1* were more variable and did not reach statistical significance. Interestingly, addition of the pool of inflammatory cytokines failed to induce activation of the AHR pathway on βlox-5 cells, where higher expression levels were observed only in the presence of NV10 or MEMB5, highlighting the specificity of the results obtained with the bacterial components (**Figures 1E–H**).

Once AHR is active, it is negatively regulated by binding of AHRR or TIPARP (TCDD-induced poly-ADP-ribose isomerase). It was observed that addition of NV10 significantly increased the expression of both repressors in presence or absence of the pool of cytokines (**Figures 1G, H**).

### NVs From *L. johnsonii* Induce AHR Translocation in Pancreatic Beta Cell Line

**Figure 2:**
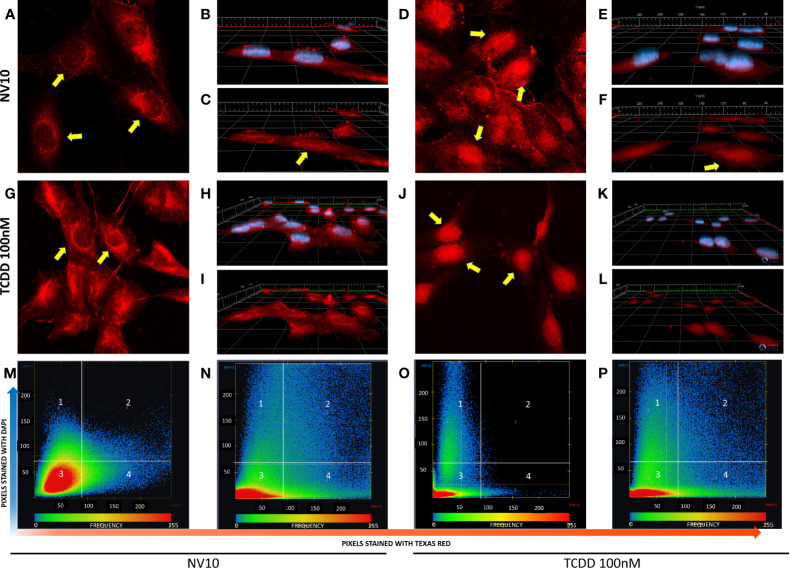
*L. johnsonii*-derived nanovesicles promote AHR nuclear translocation in βlox5 cells. Cells were incubated with NV10 **(A–F)** or 100 nM TCDD **(G–L)** and visualized by confocal microscopy. Cells were fixed and incubated with rabbit anti-AHR antibody tagged with PE-Texas red (red pixels) and the nucleus was stained with DAPI (blue pixels) as described in the methods section. **(A, G)** Time 0 (2D image) and **(B, H)** 3D z-stack image showing both stains or **(C, I)** AHR only channel. **(D, J)** 2D image, **(E, K)** 3D z-stack image showing both stains, **(F, L)** AHR only channel after 45 minutes of incubation. Yellow arrows indicate the localization of AHR in the cytoplasm and around the nuclei at time 0 and inside the nuclei after 45 minutes of incubation. **(M–P)** Heatmap showing the colocalization of both stains (DAPI and Texas red) at time 0 **(M, O)** and 45 minutes **(N, P)**, when cells were treated with NVs **(M, N)** or TCDD **(O, P)**. The upper right quadrant (number 2) shows the pixels that are stained with both stains simultaneously. The figures shown are representative of at least three biological triplicates. TCDD denotes 2,3,7,8–tetrachlorodibenzo-p-dioxin; NV10 denotes nanovesicles from *L. johnsonii* at 10^10^ particles/ml.

Activation of the canonical AHR pathway observed in presence of NVs was evaluated following the nuclear translocation of AHR, *via* immunofluorescence. As a positive control, cells were also incubated with 100 nM of TCDD, a well-known AHR inducer (27). To confirm the localization of AHR inside the nucleus, we analyzed the colocalization of both stains, Texas red (staining the AHR) and DAPI (staining the nucleus). It was observed that βlox5 cells incubated with NV10 showed an increase in the AHR nuclear translocation after 45 minutes of incubation (**Figures 2A–F**). The AHR nuclear translocation observed with NV10 treatment was comparable to the translocation observed when cells were incubated with 100 nM of TCDD (**Figures 2G–L**). For both treatments, a significant increase in the colocalization levels was observed in βlox5 cells after 45 min of incubation (**Figures 2M–P**). The colocalization coefficient increased from 0.08 to 0.18 when cells were incubated with TCDD, whereas the colocalization coefficient increased from 0.02 to 0.11 when cells were incubated with NVs (**Figures 2M–P**).

### NVs From *L. johnsonii* Induce the Expression of Genes Related to Glucose Transport and Enhance Insulin Secretion From Human Pancreatic Islets

**Figure 3:**
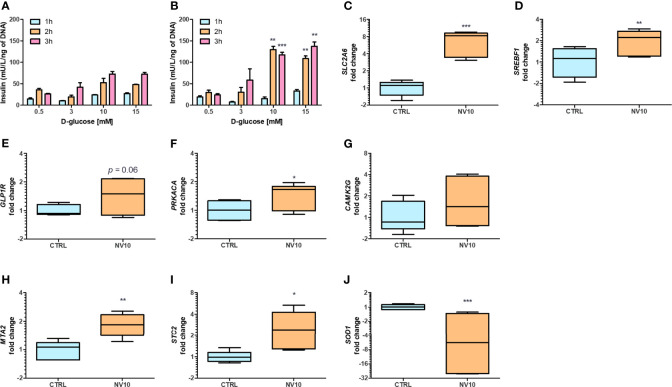
Nanovesicles from *Lactobacillus johnsonii* stimulates insulin secretion in human islets. **(A, B)** Glucose Stimulated Insulin Secretion (GSIS) was performed in human islets in absence **(A)** or presence **(B)** of NV10 with increasing concentrations of glucose (0.5, 3, 10 or 15 mM), and insulin secretion was determined in the supernatant after 1, 2 and 3 h. Insulin levels were determined by ELISA and normalized to the respective islets’ DNA concentration. **(C–J)** mRNA levels of genes involved in glucose uptake and insulin secretion pathways were evaluated in presence or absence of NVs. Human islets were incubated with NVs from *L. johnsonii* for 5 h and mRNA levels were analyzed *via* qRT-PCR. Expression levels of *SLC2A6*, *SREBF1*, *GLP1R*, *PRKACA*, *CAMK2G*, *MTA2*, *STC2*, and *SOD1*, were normalized to *GAPDH*. CTRL denotes the vehicle control group; NV10 denotes nanovesicles from *L. johnsonii* at 10^10^ particles/ml. *p < 0.05, **p < 0.01, ***p < 0.001. Data is shown as mean ± SD of at least three biological triplicates and technical duplicates.

While we observed effects from NVs in different pathways, we could not evaluate their activities on insulin secretion in βlox5 cells due to their inability to produce insulin. Expression of the gene encoding insulin (INS), and the key regulator of the transcription of insulin, the pancreatic and duodenal homeobox 1 (PDX1, also known as insulin promoter factor 1), are not expressed in βlox5 cells (28). To investigate the effect of NVs from *L. johnsonii* on insulin secretion by pancreatic beta cells, we evaluated the levels of insulin secreted by primary pancreatic islets isolated from human donors. Under starvation conditions (0.5 mM of D-glucose), there was no significant difference between the insulin levels from islets incubated with NVs when compared to the vehicle control group (**Figures 3A, B**). In presence of 3 mM D-glucose, insulin levels increased over time in both treatment groups, with more insulin being released when cells were incubated with NVs (**Figures 3A, B**). The highest concentration of insulin was observed when islets were cultured under stimulating glucose concentrations (10 or 15 mM), after 2 or 3 h in presence of NVs. These results indicate that NVs from *L. johnsonii* may be able to either increase glucose uptake in human pancreatic islets or stimulate insulin secretion over time.

The expression of genes involved in glucose uptake, as well as regulation of insulin synthesis and secretion, were analyzed on pancreatic islets. Islets treated with NV10 showed an 8-fold increase in the expression of glucose transporter Solute Carrier Family 2, Member 6 (*SLC2A6)*, also known as glucose transporter 6 (GLUT6) (**Figure 3C**), and a 2-fold increase in the Sterol Regulatory Element Binding Transcription Factor 1 (*SREBF1*) (**Figure 3D**), while expression of *SLC2A1*, *PDX1*, and *INS* were not affected after 5 h of incubation (data not shown). Glucagon-like peptide 1 receptor (GLP1R) mRNA levels were elevated in islets incubated with NVs (*p*=0.06) (**Figure 3E**). Interestingly, the expression of protein kinase catalytic subunit α (*PRKACA*), which is part of the cAMP insulin signaling pathway, was significantly increased in islets treated with NVs (**Figure 3F**). To investigate the role of NVs in insulin secretion, *via* Ca^2+^ modulation, we evaluated the mRNA levels of calcium/calmodulin dependent protein kinase II gamma (CAMK2G), where NVs were found to induce a slight increase in CAMK2G expression, however, these values did not reach statistical significance (**Figure 3G**). Altogether, these results indicate that under high glucose stimulation, NVs can induce glucose uptake by the pancreatic islets, thereby increasing insulin secretion.

### NVs From *L. johnsonii* Activate the Non-Canonical AHR Pathway Leading to Oxidative Stress Protection in Human Pancreatic Islets

Previous studies have suggested glucose-dependent insulin secretion may be directly linked to reactive oxygen species (ROS) and oxidative stress in pancreatic beta cells (29). Beta cells are inherently susceptible to oxidative stress due to high endogenous production of ROS combined with low expression levels of antioxidative enzymes (30). Metastasis Associated 1 Family Member 2 (MTA2) and Stanniocalcin-2 (STC2) play a central role in protection against oxidative stress. Upon AHR activation, MTA2 is recruited to the STC2 promoter, resulting in histone acetylation and activation of STC2 transcription, where STC2 subsequently attenuates reticulum oxidative stress and stress-induced apoptosis (31). Therefore, we evaluated the effects of NVs from *L. johnsonii* on the expression levels of genes involved in antioxidant responses. In agreement with these reports, it was found that MTA2 and STC2 mRNA levels were significantly induced (p <0.05) following treatment with NV10 (**Figures 3H, I**). To evaluate the role of NVs on oxidative stress markers, the mRNA levels of SOD1 was also determined, where NVs were found to significantly repress the expression of *SOD1* (**Figure 3J**). Taken together, these findings suggest that NVs from *L. johnsonii* may prevent intra-islet oxidative stress.

### NV Induce Markers of AHR Activation in Caco-2 Epithelial Cell and Macrophages but not in Jurkat T Cells

To evaluate whether activation of AHR by NVs is tissue specific, we tested their effects on different cell lines. THP-1 macrophages treated with NV10 showed a significant increase in the mRNA levels of *CYP1A1*, while the expression of *CYP1B1* and *AHRR* was not affected by the addition of NVs (**Supplementary Figures 2A–C**). Similarly, in Caco-2 cells, the expression of *CYP1A1* was significantly induced, while the expression of *CYP1B1* and *AHRR* was not detected after 5 hours of incubation (**Supplementary Figure 2D**). In contrast, Jurkat T cells did not show significant changes in the expression of *CYP1A1* and *CYP1B1* after 5h of incubation with NVs (**Supplementary Figures 2E, F**). In summary, the results obtained suggest that NVs differentially affect host cell types either through specific cell receptors that facilitate entry to the host cell or through regulating expression levels of target host proteins.

### NV-Mediated AHR Activation Modulates Macrophages Towards a Tolerogenic M2b Profile

**Figure 4:**
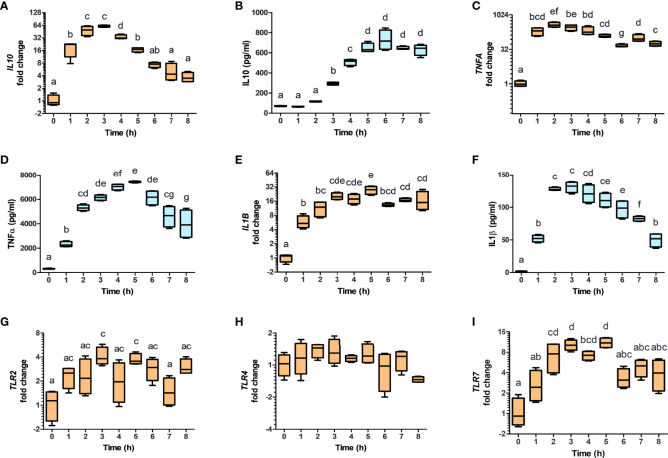
Nanovesicles from *Lactobacillus johnsonii* shift macrophages towards a M2b profile. PMA stimulated THP-1 macrophages were incubated with NVs, cytokines and mRNA (**A, C, E, G, H, I**, orange box plots) and protein levels (**B, D, F**, blue box plots) were evaluated for 8 h. Cytokine concentrations were determined in the supernatants via ELISA, while mRNA levels were determined via qRT-PCR. The mRNA levels of *IL10* **(A)**, *TNFA* **(C)**, *IL1B* **(E)**, *TLR2* **(G)**, *TLR4* **(H)** and *TLR7* **(I)**, were normalized to *GAPDH*, whereas the protein levels of IL-10 **(B)**, TNFa **(D)** and IL-1b **(F)** were normalized to the total protein concentration. Data is shown as fold change ± SD with respect to Time 0. Data shown represents at least three biological triplicates and technical duplicates. Letters on top of each group represents statistical significance (p < 0.05), where bars sharing at least one letter are not significantly different.

It has been reported that AHR activation in murine macrophages results in the polarization towards the tolerogenic M2b phenotype, through the production of IL10 (32). To evaluate the impact of NV addition on THP-1 polarization, THP-1 cells were incubated in presence or absence of NV10 for 3 and 8 h, and subsequently used to determine the mRNA and protein levels of markers of M1 or M2 phenotypes (TNFα, IL-1β, and IL-10 as well as the mRNA levels of TLR2, TLR3, TLR4, TLR7, and TLR9). We observed that cells incubated with NV10 significantly increased the mRNA and protein levels of TNFα, IL-1β and IL10 over time (**Figures 4A–F**). These results are indicative of the polarization of macrophages towards the M2b tolerogenic phenotype. In agreement with these observations, the transcription levels of TLR2 and TLR7 were significantly increased after 3 h of NV treatment while the mRNA levels of TLR4 were not affected after 8 h (**Figures 4G–I**). These data suggest that NVs from *L. johnsonii* can induce a shift in the phenotype of macrophages towards an anti-inflammatory profile.

**Figure 5:**
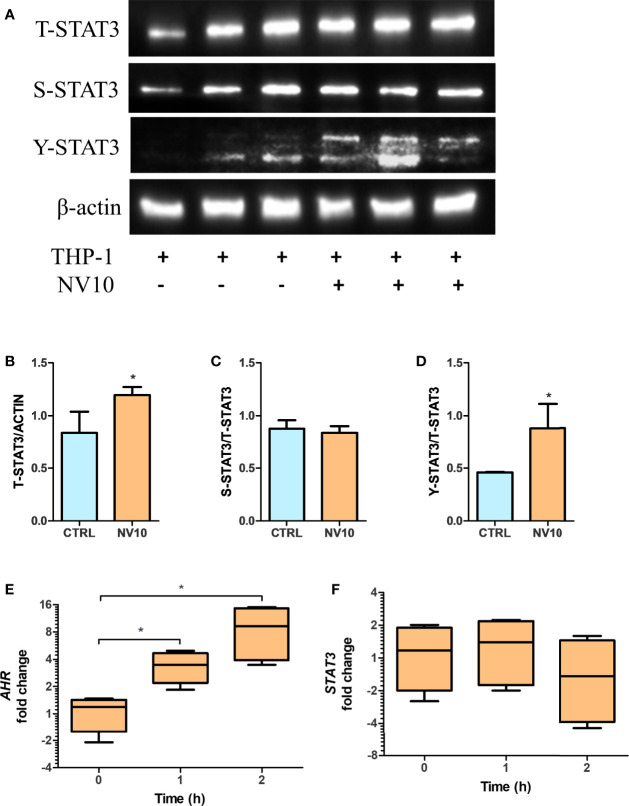
*Lactobacillus johnsonii-*derived nanovesicles alter STAT3 phosphorylation and AHR mRNA levels in THP-1 macrophages. THP-1 cells were treated with vehicle control (blue bars) or *L. johnsonii*-derived nanovesicles (orange bars) for 5 h. **(A)** Western blot showing total STAT3 (T-STAT3), and phosphorylation at S727 (S-STAT3) and Y705 (Y-STAT3). β-actin was used as a loading control. Three biological replicates are shown for each treatment as indicated. **(B–D)** Quantification of band intensity was performed with ImageJ and expressed as **(B)** T-STAT3/ACTIN, **(C)** S-STAT3/T-STAT3 and **(D)** Y-STAT3/T-STAT3 ratios. **(E, F)** The mRNA levels of *AHR* **(E)** and *STAT3* **(F)** were evaluated under similar conditions *via* qRT-PCR. CTRL denotes vehicle control group; NV10 denotes nanovesicles from *L. johnsonii* at 10^10^ particles/ml. Data are expressed as mean ± SD. *p < 0.05.

The mRNA expression of IL10 is controlled by multiple transcription factors including STAT3 and AHR (32). To determine the involvement of the STAT3 pathway in NV-induced IL10 expression, the activation of STAT3 was quantified by of S727 and Y705 phosphorylation (pS-STAT3 and pY-STAT3, respectively) as well as total STAT3 (T-STAT3) in THP-1 cells exposed to NV10. It was found that addition of NV10 resulted in a significant increase in the T-STAT3/actin ratio as well as pY-STAT3/T-STAT3, while pS-STAT3/T-STAT3 was not affected (**Figures 5A–D**). These results indicate that the addition of NVs promote the transcriptional activity of STAT3 as Y705 phosphorylation is required for the role of STAT3 as a transcription factor and binding to specific promoters in the nucleus (33). It was also observed that the increased pY-STAT3 positively correlated with a significant increase in the mRNA expression of AHR (**Figures 5E, F**). These results indicate that the induced expression of the CYP genes is consequence of an indirect effect of NVs on the AHR pathway, and that STAT3 is the primary target of NVs in macrophages. However, the observed increase in expression of *IL10* may be explained by the combined function of STAT3 as well as AHR, since binding sites for both transcription factors have been reported to activate the *IL10* promoter (32).

### Stimulation of THP-1 With NVs Increase IL-10 Expression in βlox5 *via* STAT3 Activation

**Figure 6:**
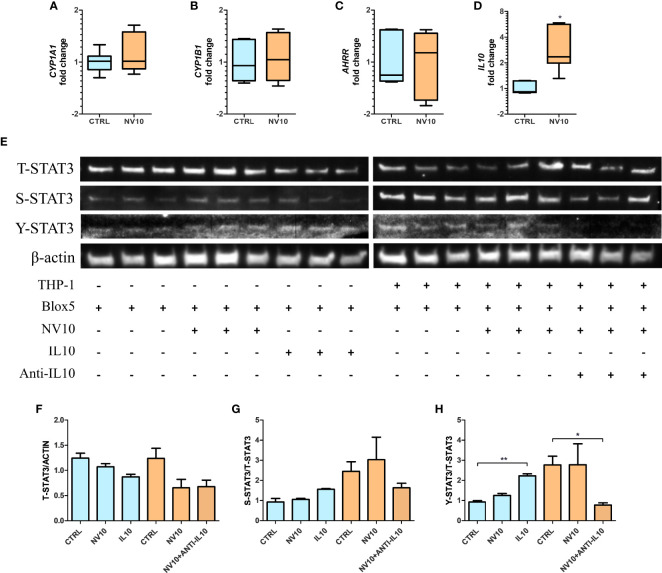
Stimulation of THP-1 with nanovesicles from *Lactobacillus johnsonii* increases IL-10 expression in βlox5 *via* STAT3 activation. Co-culture of βlox5 and THP-1 cells were performed as described in the Materials and methods sections. **(A–D)**: blue bars represent the vehicle control group, whereas orange bar represents the NV10 treatment group. The mRNA levels of *CYP1A1* **(A)**, *CYP1B1* **(B)**, *AHRR* **(C)** and *IL10* **(D)** were evaluated on βlox5 cells after co-cultured with THP-1 cells *via* qRT-PCR. Gene expression is shown relative to the vehicle control. **(E)** Western blot showing the total STAT3 (T-STAT3) and phosphorylation at S727 (S-STAT3) and Y705 (Y-STAT3). Three biological replicates are shown for each treatment as indicated. **(F–H)** Quantification of band intensity was performed with ImageJ and expressed as **(F)** T-STAT3/ACTIN, **(G)** S-STAT3/T-STAT3 and **(H)** Y-STAT3/STAT3 ratios. Blue bars represent the protein levels in βlox5 in the absence of THP-1, while the orange bars represent the protein levels in Blox5 after being co-cultured with THP-1 cells. CTRL denotes vehicle control group; NV10 denotes nanovesicles from *L. johnsonii* at 10^10^ particles/ml. Data are expressed as mean ± SD. *p < 0.05; **p < 0.01.

Macrophages have an important role in the pancreatic tissue, sensing danger associated molecular patterns (DAMPs) released by injured tissue, resulting in the activation of an inflammatory response. Alternatively, they can act as mediators of anti-inflammatory responses through the secretion of IL10 (34). Therefore, we determined the effects of the cytokines secreted by NV-treated THP-1 cells on βlox5 cells. Inserts containing 6x10^5^ PMA-activated THP-1 cells were transferred to 6-well plates containing 6x10^5^ βlox5 cells/well. Stimulation was performed by addition of NVs at 10^10^ particles/mL to the top chamber of the inserts (empty inserts or PMA-activated THP-1 cells), and the expression of AHR activation markers was quantified after 5 h. It was found that treatment of THP-1 cells with NVs did not significantly affect the expression of *CYP1A1*, *CYP1B1* and *AHRR* in βlox5 (**Figures 6A–C**). In contrast, the expression of IL10 mRNA was significantly increased in βlox5 cells that were co-cultured with THP-1 (**Figure 6D**). These results are significant since our transcriptome results showed that the IL10 gene is not expressed in βlox5 cells in absence of THP-1 cells.

These results suggest that different mechanisms are involved in NV signaling depending on the cell lines tested and/or cells in their environment within a tissue. We hypothesize that THP-1-secreted IL10 acts as the signaling molecule that stimulates the STAT3 pathway in βlox5 cells resulting in further IL10 expression. First, the levels of T-STAT3 as well as S-STAT3 and Y-STAT3 were evaluated in βlox5 cells after 5 h of co-culture with THP-1, in presence or absence of NV10 (**Figure 6E**) as described above. The stimulation of βlox5 cells with NV10 in absence of THP-1 cells did not significantly affect the levels of T-, S- or Y-STAT3 (**Figures 6F–H**). Interestingly, the co-culture of THP-1 with βlox5 cells resulted in a significant increase in Y-STAT3 phosphorylation, while further stimulation was not observed upon addition of NVs to THP-1. The levels of S-STAT3 were not affected under any conditions tested.

To evaluate whether IL10 secretion by THP1 is involved in STAT3 activation in βlox5 cells, IL10 at 100 ng/ml was added to an empty insert, and anti-IL-10 at 1µg/ml was added to a co-culture containing NV-stimulated THP-1 and βlox5 cells, where the levels of Y-STAT3 were significantly increased in βlox5 cells in presence of IL10. In contrast, levels of Y-STAT3 were significantly decreased following neutralization of IL10 with monoclonal antibodies (**Figures 6E–H**).

## Discussion

**Figure 7:**
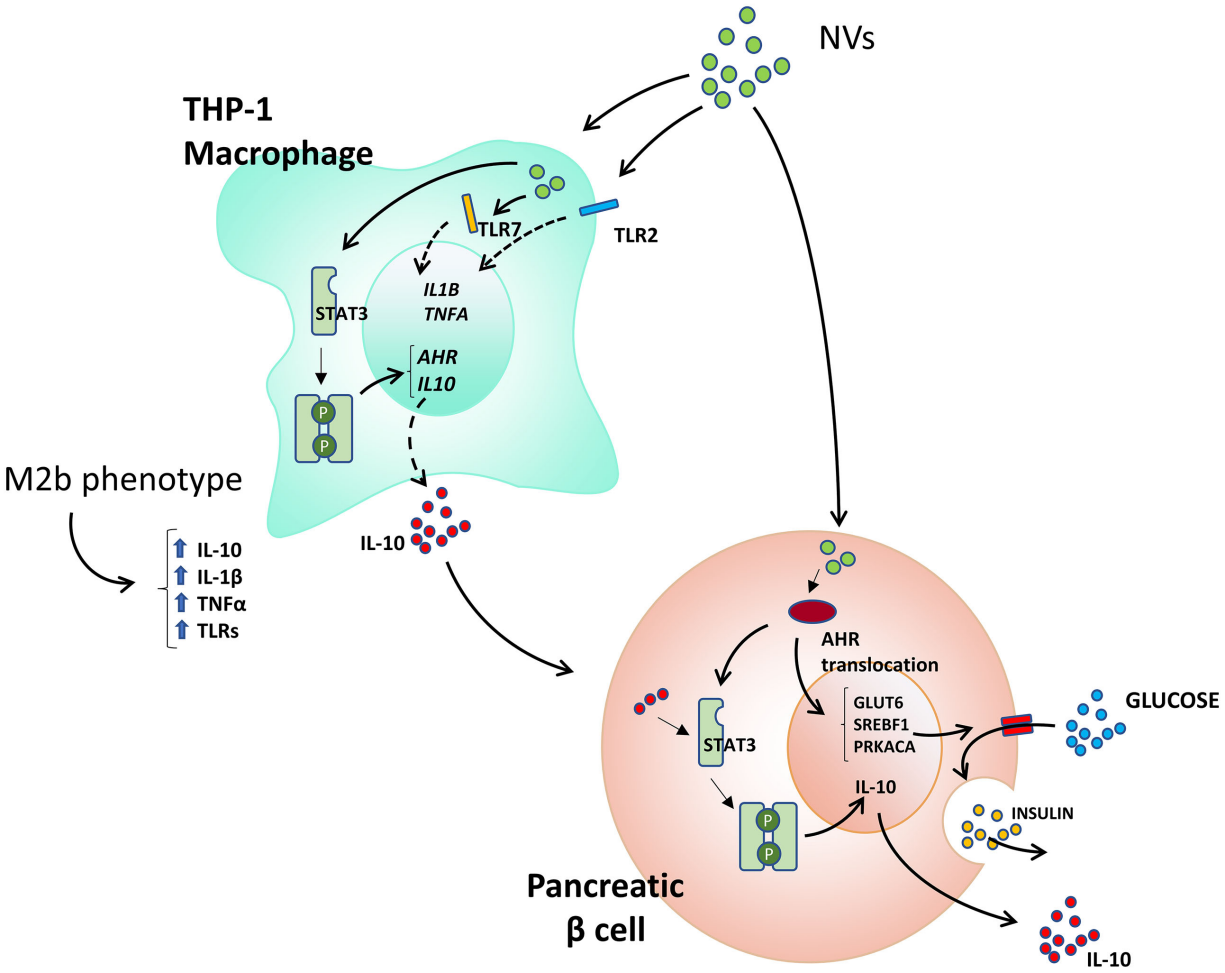
Proposed mechanisms of action of *L. johnsonii*-derived NVs on macrophage and beta cells.

The role of extracellular vesicles in cell-to-cell communication in eukaryotic cells has been broadly studied [recently reviewed in (35)] however, the impact of bacterial vesicles in cross kingdom communication is emerging as a fascinating mechanism of bacterial activity at locations distant to infection. The results obtained in this work show that NVs secreted by *L. johnsonii* have a selective impact on eukaryotic cell lines as well as human pancreatic islets. We found that NVs can mediate AHR activation, protecting pancreatic beta cells from apoptosis and promoting insulin secretion under high glucose stimulation as well as inducing a shift of M1 macrophages towards a tolerogenic M2 profile (**Figure 7**). While the AHR signaling pathway has been reported to play a pivotal role in health and disease, the role of bacterial extracellular vesicles as inducers of AHR nuclear translocation has not been previously reported in the context of type 1 diabetes.

Previously, we showed that *L. johnsonii* was able to delay onset of type 1 diabetes when administered orally to BB-DP rats. Administration of *L. johnsonii* regulated multiple inflammatory pathways, including IDO, the inflammasome, and mTOR pathways (8, 10, 11, 19). Although we have consistently observed the anti-inflammatory properties of *L. johnsonii* both *in vivo* and *in vitro*, the mechanism of action is still not completely understood. Most recently, we found that *L. johnsonii* can produce extracellular, nano-sized vesicles (NVs). Their proteomic and lipidomic profile indicated differential enrichment of protein and lipid signatures when compared to the membrane of the whole bacterium (15). Based on these findings, we hypothesized that *L. johnsonii* NVs are carriers of bioactive molecules that can modulate the responses of pancreatic beta cells to an exacerbated macrophage or T cell immune response. Here we show that *L. johnsonii* NVs were able to decrease apoptosis of human beta cells (**Figure 1**). These results are significant since beta cell death is an important hallmark of diabetes onset. The role of probiotic-derived vesicles on apoptosis rates has been recently reported in the context of colorectal cancer, where *Lacticaseibacillus paracasei* PC-H1 extracellular vesicles were reported to inhibit phosphorylation of 3-phosphoinisitide-dependent protein kinase (PDK1) and AKT and reduced the expression of Bcl-2 protein (36). In a model of type 2 diabetes, a mix of live probiotics were able to reduce the expression of pro-apoptotic Bcl-2 and Caspase 3, while increasing levels of the anti-apoptotic protein Bax and inducing the PI3K/AKT pathway (37). These results are interesting and highlight the different mechanisms employed by specific strains of probiotic microorganism as well as their variable effects at different locations within the host. We have previously reported that *L. johnsonii* modulates the AKT/mTOR pathway in the gastrointestinal tract, but it does not affect their activation levels in the pancreas (19). The data obtained in rodent models was confirmed here with human beta cell lines as well as in human islets. Moreover, the levels of pro- or anti-apoptotic genes were not correlated with a decrease in apoptosis observed upon NV treatment of human beta cell lines. Based on these findings, we conducted unbiased RNAseq to uncover the AHR pathway as the mechanistic target of *L. johnsonii* NVs on human beta cells.

AHR is a ubiquitous ligand-activated transcriptional factor, whose activity is modulated through binding a broad variety of agonist and antagonist ligands [reviewed in (38)]. As such, AHR signaling has been recognized as a key regulator of several immune diseases, however, current literature regarding the impact of AHR activation on the pathogenesis of type 1 diabetes suggests that its effect is indirect through immune modulation and not directly on beta cells. As an example, administration of the AHR agonist TCDD prevented diabetes onset in NOD mice by increasing the frequency of Foxp3+T cells in pancreatic lymph nodes (39). In agreement with this finding, in other models of autoimmunity, AHR activation with 6-formylindolo[3,2-b] carbazole (FICZ) and 2,(1’H’indole, 3’ carbonyl) thiazole-4-carboxylic acid methyl ester (ITE) was correlated with reduced dendritic cell antigen presentation, as well as T_H_1 and T_H_17 cell activation (40). Our results show that *L. johnsonii* NVs induced activation and nuclear translocation of AHR in human beta cells, resulting in stimulation of insulin release in human islets under high glucose stimulation. The role of probiotic-derived vesicles on AHR activation has been reported recently in intestinal tissue by exosome-like nanoparticles from *Lactobacillus rhamnosus* GG (LDNPs) (41). It was shown that oral administration of the vesicles was able to increase mRNA levels of *CYP1A1*, a marker of AHR activation, in the ilium of BALB/c mice. The authors proposed that activation of AHR is through the metabolites indoleacrylic acid (IA) and indole-3-aldehyde present in the LDNPs. Interestingly, these metabolites were reported as inducers of AHR by whole cells of *Peptostreptococcus* species, suppressing inflammation in both epithelial cells and macrophages (42). Moreover, the addition of pure IA resulted in stimulation of IL10 secretion under LPS-stimulated macrophages. It is possible that the effect observed with *L. johnsonii*-derived NVs on beta cells is mediated by metabolites found inside the NVs. Another possibility is that bacterial proteins contained within the NVs mediate this activity through protein-protein interactions. Our results indicate that *L. johnsonii*-derived NVs modulate the activity of AHR as well as its mRNA levels, indicative of additional mechanisms of activity that may not be explained through metabolite activation of AHR. Alternatively, it has been reported that IL10 mRNA levels can be regulated through binding of several transcription factors, such as AHR and STAT3 (32, 43). Here we found that *L. johnsonii*-derived NVs promote phosphorylation of STAT3 at tyrosine 705 (Y705) in both THP1 macrophages and beta cells, resulting in increased mRNA levels of AHR. These results agree with observations that increased levels of AHR can promote the expression of *IL10 via* a non-canonical pathway (independent of nuclear translocation). Through this pathway, high levels of AHR in the cytoplasm can induce the activation of STAT3 by the phosphorylation of Y705 triggered by Src tyrosine kinase (32).

A recent report indicated the role of microbial proteins in activation of STAT3 phosphorylation followed by nuclear translocation. To escape from an inflammatory response, *Salmonella* infections trigger the expression of IL10, reducing the expression of pro-inflammatory cytokines, as well as the reduction of ROS secretion by macrophages and inhibition of caspase-1 activation (44). It was found that the *Salmonella anti-inflammatory response activator* (*sarA*), secreted *via* the type 3 secretion system, strongly induce the secretion of IL10 by lymphoblastoid cell lines (LCLs) (45). Furthermore, *sarA* stimulated *Il10* transcription by STAT3 activation *via* the pY705. We hypothesize that a protein contained on the surface or inside the *L. johnsonii* NVs mediates pY-STAT3 activation following a similar mechanism as SarA, however, a genomic search failed to identify a SarA homolog in *L. johnsonii*.

Another interesting result of this study is the significant increase in the IL-10 expression observed when βlox5 cells were co-cultured with NV-treated THP-1 cells, considering that only NVs were not able to induce βlox5 cells to express *IL10.* Similarly, we identified an increase in the phosphorylation levels of STAT3 when βlox5 cells were co-incubated with THP-1 cells. These results suggest that IL-10 secreted by THP-1 cells is sensed by IL10 receptors on βlox5 cell membranes, triggering the phosphorylation of STAT3 and, consequently, transcription of *IL10*. This signaling pathway has been previously implicated in type 1 diabetes prevention in the NOD model, where it was shown that administration of IL-10 or viral IL-10 gene transfer were effective in the prevention of autoimmune diabetes in NOD mice (46–48). The limitation of our study is that the effect of NVs has not been evaluated *in vivo*. Further experiments are required to address the optimal route and dosage of NVs for a preventive treatment of type 1 diabetes.

The role of NVs on AHR activation were positively correlated with a significant increase in pancreatic beta cell function. The increased insulin secretion correlated with increased expression of the glucose transporter *SLC2A6* (*GLUT6*). Glucose uptake mediated by glucose transporters are an essential step in the glucose-dependent insulin secretion pathway (49). *SLC2A6* is expressed in all cell types of the pancreatic islets and the knockdown of *SLC2A6* significantly reduces glucose metabolism and induce cell death (49). We also observed a significant increase in the expression levels of *SREBF1*, which has been shown to be involved in insulin secretion in pancreatic beta cells. Mutations in *SREBF1* sequence are associated with an increase in type 2 diabetes risk and insulin resistance (50). Our data suggest that NVs increase both glucose uptake and insulin secretion in pancreatic islets. The observation that *L. johnsonii* NVs increased insulin secretion only at high glucose levels shows that NVs may potentially have a protective effect against insulin resistance and in type 2 diabetes metabolic syndrome.

Altogether, our results showed that NVs from *L. johnsonii* have the potential to regulate the intra-islet environment, inducing a strong anti-inflammatory response that may ameliorate type 1 diabetes symptoms *in vivo*.

## Data Availability Statement

The datasets presented in this study can be found in online repositories. The name of the repository and accession number can be found below: https://www.ncbi.nlm.nih.gov/bioproject/PRJNA804404.

## Author Contributions

LT, NH, and DS performed the experiments. LT, CG, CM, and GL contributed to the conception and design of the study. LT analyzed the data. LT wrote the original draft. LT, CG, CM and GL reviewed and edited the manuscript. All authors have read and agreed to the published version of the manuscript.

## Funding

This study was funded by the National Institute of Diabetes and Digestive and Kidney Diseases of the National Institutes of Health under award number R01DK121130 and R01DK127497, and by the National Institute of Allergy and Infectious Diseases of the National Institutes of Health under award number P01AI042288. The content is solely the responsibility of the authors and does not necessarily represent the official views of the National Institutes of Health.

## Conflict of Interest

GL holds U.S. patent No. 9,474,773 and 9,987,313 on *Lactobacillus johnsonii* N6.2.

The remaining authors declare that the research was conducted in the absence of any commercial or financial relationships that could be constructed as a potential conflict of interest.

## Publisher’s Note

All claims expressed in this article are solely those of the authors and do not necessarily represent those of their affiliated organizations, or those of the publisher, the editors and the reviewers. Any product that may be evaluated in this article, or claim that may be made by its manufacturer, is not guaranteed or endorsed by the publisher.

## References

[1] Atkinson MA Eisenbarth GS Michels AW. Type 1 Diabetes. Lancet (2014) 383:69–82. doi: 10.1016/S0140-6736(13)60591-7 23890997 PMC4380133

[2] Mejía-León ME López-Domínguez L Aguayo-Patrón SV Caire-Juvera G Calderón de la Barca AM. Dietary Changes and Gut Dysbiosis in Children With Type 1 Diabetes. J Am Coll Nutr (2018) 37:501–7. doi: 10.1080/07315724.2018.1444519 29634398

[3] Stewart CJ Ajami NJ O’Brien JL Hutchinson DS Smith DP Wong MC. Temporal Development of the Gut Microbiome in Early Childhood From the TEDDY Study. Nature (2018) 562:583–8. doi: 10.1038/s41586-018-0617-x PMC6415775 30356187

[4] Uusitalo U Lee H-S Andrén Aronsson C Vehik K Yang J Hummel S. Early Infant Diet and Islet Autoimmunity in the TEDDY Study. Diabetes Care (2018) 41:522–30. doi: 10.2337/dc17-1983 PMC5829968 29343517

[5] Fuller R. Probiotics in Man and Animals. J Appl Bacteriol (1989) 66:365–78. doi: 10.1111/j.1365-2672.1989.tb05105.x 2666378

[6] Swanson KS Gibson GR Hutkins R Reimer RA Reid G Verbeke K. The International Scientific Association for Probiotics and Prebiotics (ISAPP) Consensus Statement on the Definition and Scope of Synbiotics. Nat Rev Gastroenterol Hepatol (2020) 17:687–701. doi: 10.1038/s41575-020-0344-2 32826966 PMC7581511

[7] Roesch LF Lorca GL Casella G Giongo A Naranjo A Pionzio AM. Culture-Independent Identification of Gut Bacteria Correlated With the Onset of Diabetes in a Rat Model. ISME J (2009) 3:536. doi: 10.1038/ISMEJ.2009.5 19225551 PMC2972309

[8] Valladares R Sankar D Li N Williams E Lai K-K Abdelgeliel AS. Lactobacillus Johnsonii N6.2 Mitigates the Development of Type 1 Diabetes in BB-DP Rats. PLoS One (2010) 5:e10507. doi: 10.1371/journal.pone.0010507 20463897 PMC2865539

[9] Lau K Benitez P Ardissone A Wilson TD Collins EL Lorca G. Inhibition of Type 1 Diabetes Correlated to a *Lactobacillus johnsonii* N6.2-Mediated Th17 Bias. J Immunol (2011) 186:3538–46. doi: 10.4049/jimmunol.1001864 21317395

[10] Valladares R Bojilova L Potts AH Cameron E Gardner C Lorca G. *Lactobacillus johnsonii* Inhibits Indoleamine 2,3-Dioxygenase and Alters Tryptophan Metabolite Levels in BioBreeding Rats. FASEB J (2013) 27:1711–20. doi: 10.1096/fj.12-223339 23303207

[11] Teixeira LD Kling DN Lorca GL Gonzalez CF. Lactobacillus Johnsonii N6.2 Diminishes Caspase-1 Maturation in the Gastrointestinal System of Diabetes Prone Rats. Benef Microbes (2018) 9:527–39. doi: 10.3920/BM2017.0120 29633641

[12] Liu H Xu R Kong Q Liu J Yu Z Zhao C. Downregulated NLRP3 and NLRP1 Inflammasomes Signaling Pathways in the Development and Progression of Type 1 Diabetes Mellitus. BioMed Pharmacother (2017) 94:619–26. doi: 10.1016/j.biopha.2017.07.102 28783585

[13] Marcial GE Ford AL Haller MJ Gezan SA Harrison NA Cai D. Lactobacillus Johnsonii N6.2 Modulates the Host Immune Responses: A Double-Blind, Randomized Trial in Healthy Adults. Front Immunol (2017) 8:655. doi: 10.3389/fimmu.2017.00655 28659913 PMC5466969

[14] Yasui H Shida K Matsuzaki T Yokokura T. Immunomodulatory Function of Lactic Acid Bacteria. Antonie Van Leeuwenhoek (1999) 76:383–9. doi: 10.1007/978-94-017-2027-4_24 10532394

[15] Harrison NA Gardner CL da Silva DR Gonzalez CF Lorca GL. Identification of Biomarkers for Systemic Distribution of Nanovesicles From Lactobacillus Johnsonii N6.2. Front Immunol (2021) 12:723433. doi: 10.3389/fimmu.2021.723433 34531870 PMC8438180

[16] Briaud P Carroll RK. Extracellular Vesicle Biogenesis and Functions in Gram-Positive Bacteria. Infect Immun (2020) 88(12):e00433–20. doi: 10.1128/IAI.00433-20 PMC7671900 32989035

[17] Love MI Huber W Anders S. Moderated Estimation of Fold Change and Dispersion for RNA-Seq Data With Deseq2. Genome Biol (2014) 15:550. doi: 10.1186/s13059-014-0550-8 25516281 PMC4302049

[18] Halvorsen TL Leibowitz G Levine F. Telomerase Activity Is Sufficient To Allow Transformed Cells To Escape From Crisis. Mol Cell Biol (1999) 19:1864–70. doi: 10.1128/MCB.19.3.1864 PMC83979 10022873

[19] Kling DN DeBose-Scarlett EM Teixeira LD Gezan SA Lorca GL Gonzalez CF. Sex Modulates Lactobacillus Johnsonii N6.2 and Phytophenol Effectiveness in Reducing High Fat Diet Induced mTOR Activation in Sprague-Dawley Rats. Front Microbiol (2018) 9:2649. doi: 10.3389/fmicb.2018.02649 30459740 PMC6232610

[20] Chen S Zhou Y Chen Y Gu J. Fastp: An Ultra-Fast All-in-One FASTQ Preprocessor. Bioinformatics (2018) 34:i884–90. doi: 10.1093/bioinformatics/bty560 PMC6129281 30423086

[21] Liao Y Smyth GK Shi W. Featurecounts: An Efficient General Purpose Program for Assigning Sequence Reads to Genomic Features. Bioinformatics (2014) 30:923–30. doi: 10.1093/bioinformatics/btt656 24227677

[22] Walter W Sánchez-Cabo F Ricote M. GOplot: An R Package for Visually Combining Expression Data With Functional Analysis: Fig. 1. Bioinformatics (2015) 31:2912–4. doi: 10.1093/bioinformatics/btv300 25964631

[23] Tomita T. Apoptosis of Pancreatic β-Cells in Type 1 Diabetes. Bosn J Basic Med Sci (2017) 17:183. doi: 10.17305/BJBMS.2017.1961 28368239 PMC5581966

[24] Sutton VR Estella E Li C Chen M Thomas HE Kay TW. Critical Role for Granzyme B, in Addition to Perforin and TNF??, in Alloreactive CTL-Induced Mouse Pancreatic Beta Cell Death. Transplantation (2006) 81:146–54. doi: 10.1097/01.tp.0000191939.68451.d9 16436955

[25] Drappier M Michiels T. Inhibition of the OAS/RNase L Pathway by Viruses. Curr Opin Virol (2015) 15:19–26. doi: 10.1016/j.coviro.2015.07.002 26231767 PMC7185432

[26] Yao EF Denison MS. DNA Sequence Determinants for Binding of Transformed Ah Receptor to a Dioxin-Responsive Enhancer. Biochemistry (1992) 31:5060–7. doi: 10.1021/bi00136a019 1318077

[27] Ehrlich AK Pennington JM Bisson WH Kolluri SK Kerkvliet NI. TCDD, FICZ, and Other High Affinity AhR Ligands Dose-Dependently Determine the Fate of CD4+ T Cell Differentiation. Toxicol Sci (2018) 161:310–20. doi: 10.1093/toxsci/kfx215 PMC5837604 29040756

[28] Itkin-Ansari P Geron I Hao E Demeterco C Tyrberg B Levine F. Cell-Based Therapies for Diabetes: Progress Towards a Transplantable Human β Cell Line. Ann N Y Acad Sci (2003) 1005:138–47. doi: 10.1196/annals.1288.015 14679048

[29] Fridlyand LE Philipson LH. Does the Glucose-Dependent Insulin Secretion Mechanism Itself Cause Oxidative Stress in Pancreatic -Cells? Diabetes (2004) 53:1942–8. doi: 10.2337/diabetes.53.8.1942 15277370

[30] Wang J Wang H. Oxidative Stress in Pancreatic Beta Cell Regeneration. Oxid Med Cell Longev (2017) 2017:91930261. doi: 10.1155/2017/1930261 PMC5560096 28845211

[31] Wajda A Łapczuk-Romańska J Paradowska-Gorycka A. Epigenetic Regulations of AhR in the Aspect of Immunomodulation. Int J Mol Sci (2020) 21:1–28. doi: 10.3390/IJMS21176404 PMC7504141 32899152

[32] Zhu J Luo L Tian L Yin S Ma X Cheng S. Aryl Hydrocarbon Receptor Promotes IL-10 Expression in Inflammatory Macrophages Through Src-STAT3 Signaling Pathway. Front Immunol (2018) 9:2033. doi: 10.3389/fimmu.2018.02033 30283437 PMC6156150

[33] Huang G Yan H Ye S Tong C Ying Q. STAT3 Phosphorylation at Tyrosine 705 and Serine 727 Differentially Regulates Mouse ESC Fates. Stem Cells (2014) 32:1149–60. doi: 10.1002/stem.1609 PMC4181708 24302476

[34] Carpenter RL Lo HW. STAT3 Target Genes Relevant to Human Cancers. Cancers (Basel) (2014) 6:897–925. doi: 10.3390/cancers6020897 24743777 PMC4074809

[35] Samanta S Rajasingh S Drosos N Zhou Z Dawn B Rajasingh J. Exosomes: New Molecular Targets of Diseases. Acta Pharmacol Sin (2018) 39:501–13. doi: 10.1038/aps.2017.162 PMC5888687 29219950

[36] Shi Y Meng L Zhang C Zhang F Fang Y. Extracellular Vesicles of Lacticaseibacillus Paracasei PC-H1 Induce Colorectal Cancer Cells Apoptosis *via* PDK1/AKT/Bcl-2 Signaling Pathway. Microbiol Res (2022) 255:126921. doi: 10.1016/j.micres.2021.126921 34839170

[37] Wang Y Dilidaxi D Wu Y Sailike J Sun X Nabi Xh. Composite Probiotics Alleviate Type 2 Diabetes by Regulating Intestinal Microbiota and Inducing GLP-1 Secretion in Db/Db Mice. BioMed Pharmacother (2020) 125:109914. doi: 10.1016/J.BIOPHA.2020.109914 32035395

[38] Carambia A Schuran FA. The Aryl Hydrocarbon Receptor in Liver Inflammation. Semin Immunopathol (2021) 43:563–75. doi: 10.1007/s00281-021-00867-8 PMC8443474 34075438

[39] Kerkvliet NI Steppan LB Vorachek W Oda S Farrer D Wong CP. Activation of Aryl Hydrocarbon Receptor by TCDD Prevents Diabetes in NOD Mice and Increases Foxp3 + T Cells in Pancreatic Lymph Nodes. Immunotherapy (2009) 1:539–47. doi: 10.2217/imt.09.24 PMC2823486 20174617

[40] Wang C Ye Z Kijlstra A Zhou Y Yang P. Activation of the Aryl Hydrocarbon Receptor Affects Activation and Function of Human Monocyte-Derived Dendritic Cells. Clin Exp Immunol (2014) 177:521–30. doi: 10.1111/cei.12352 PMC4226603 24749687

[41] Gu Z Li F Liu Y Jiang M Zhang L He L. Exosome-Like Nanoparticles From Lactobacillus rhamnosusGG Protect Against Alcohol-Associated Liver Disease Through Intestinal Aryl Hydrocarbon Receptor in Mice. Hepatol Commun (2021) 5:846. doi: 10.1002/HEP4.1679 34027273 PMC8122379

[42] Wlodarska M Luo C Kolde R D’Hennezel E Annand JW Heim CE. Indoleacrylic Acid Produced by Commensal Peptostreptococcus Species Suppresses Inflammation. Cell Host Microbe (2017) 22:25–37.e6. doi: 10.1016/j.chom.2017.06.007 28704649 PMC5672633

[43] Piper CJM Rosser EC Oleinika K Nistala K Krausgruber T Rendeiro AF. Aryl Hydrocarbon Receptor Contributes to the Transcriptional Program of IL-10-Producing Regulatory B Cells. Cell Rep (2019) 29:1878–92.e7. doi: 10.1016/j.celrep.2019.10.018 31722204 PMC6856759

[44] van Niel G D’Angelo G Raposo G. Shedding Light on the Cell Biology of Extracellular Vesicles. Nat Rev Mol Cell Biol (2018) 19:213–28. doi: 10.1038/nrm.2017.125 29339798

[45] Jaslow SL Gibbs KD Fricke WF Wang L Pittman KJ Mammel MK. Salmonella Activation of STAT3 Signaling by SarA Effector Promotes Intracellular Replication and Production of IL-10. Cell Rep (2018) 23:3525–36. doi: 10.1016/j.celrep.2018.05.072 PMC6314477 29924996

[46] Yang Z Chen M Wu R Fialkow LB Bromberg JS McDuffie M. Suppression of Autoimmune Diabetes by Viral IL-10 Gene Transfer. J Immunol (2002) 168:6479–85. doi: 10.4049/JIMMUNOL.168.12.6479 12055268

[47] Xu A Zhu W Li T Li X Cheng J Li C. Interleukin-10 Gene Transfer Into Insulin-Producing β Cells Protects Against Diabetes in non-Obese Diabetic Mice. Mol Med Rep (2015) 12:3881–9. doi: 10.3892/mmr.2015.3809 25998958

[48] Preisser TM da Cunha VP Santana MP Pereira VB Cara DC Souza BM. Recombinant Lactococcus Lactis Carrying IL-4 and IL-10 Coding Vectors Protects Against Type 1 Diabetes in NOD Mice and Attenuates Insulitis in the STZ-Induced Model. J Diabetes Res (2021) 2021:1–15. doi: 10.1155/2021/6697319 PMC7872750 33604389

[49] Segerstolpe Å Palasantza A Eliasson P Andersson EM Andréasson AC Sun X. Single-Cell Transcriptome Profiling of Human Pancreatic Islets in Health and Type 2 Diabetes. Cell Metab (2016) 24:593–607. doi: 10.1016/j.cmet.2016.08.020 27667667 PMC5069352

[50] Grarup N Stender-Petersen KL Andersson EA Jørgensen T Borch-Johnsen K Sandbæk A. Association of Variants in the Sterol Regulatory Element-Binding Factor 1 (SREBF1) Gene With Type 2 Diabetes, Glycemia, and Insulin Resistance A Study of 15,734 Danish Subjects. Diabetes (2008) 57:1136–42. doi: 10.2337/db07-1534 18192539

