# Supplementary figures and images for: Nanovesicles From Lactobacillus johnsonii N6.2 Reduce Apoptosis in Human Beta Cells by Promoting AHR Translocation and IL10 Secretion
Source: Front Immunol. 2022 Jun 9;13:899413. doi: 10.3389/fimmu.2022.899413 (PMC9221839; doi:10.3389/fimmu.2022.899413)

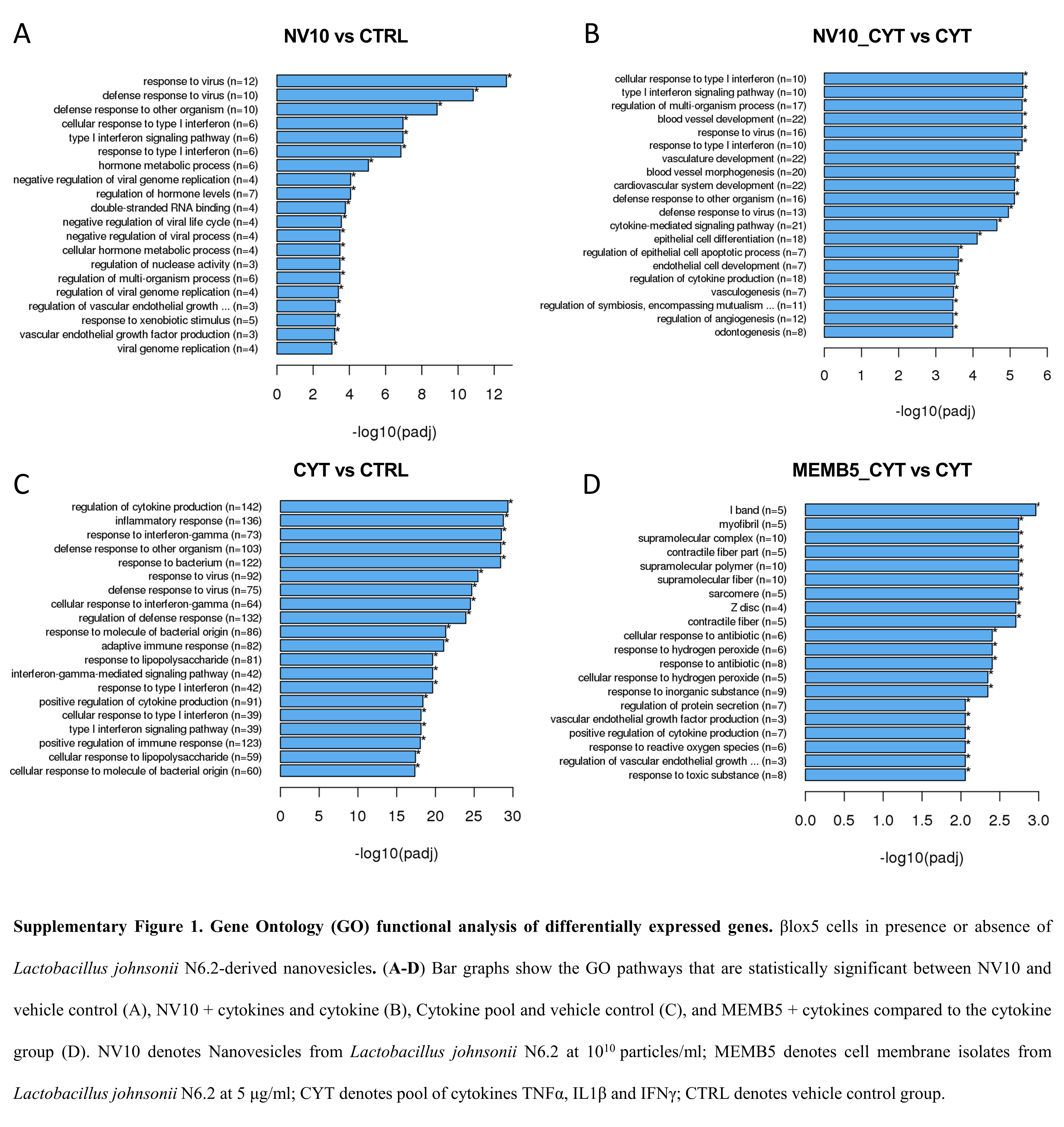

Supplement: Supplementary file 1 [file Image_1.tif]

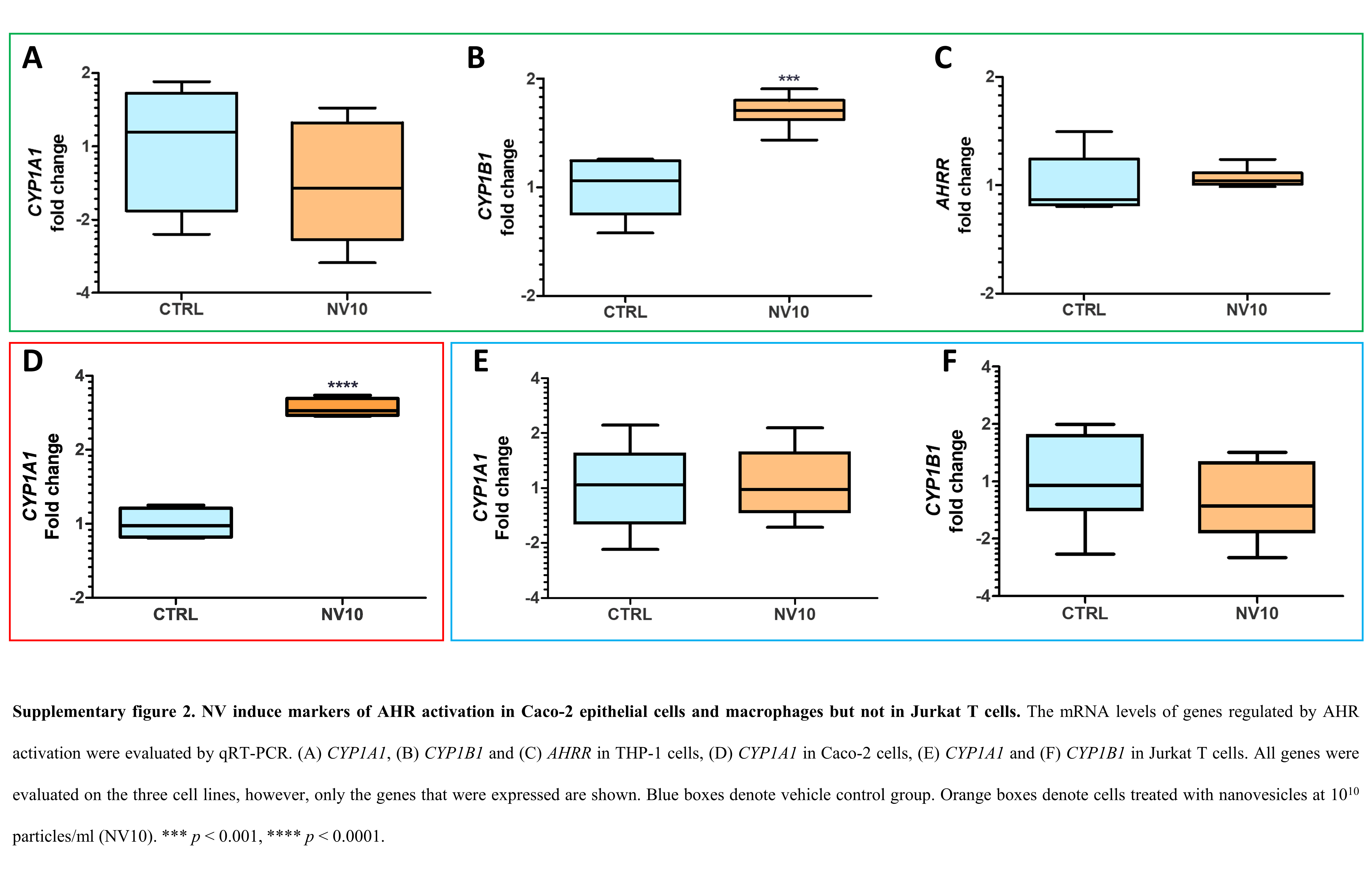

Supplement: Supplementary file 2 [file Image_2.tif]

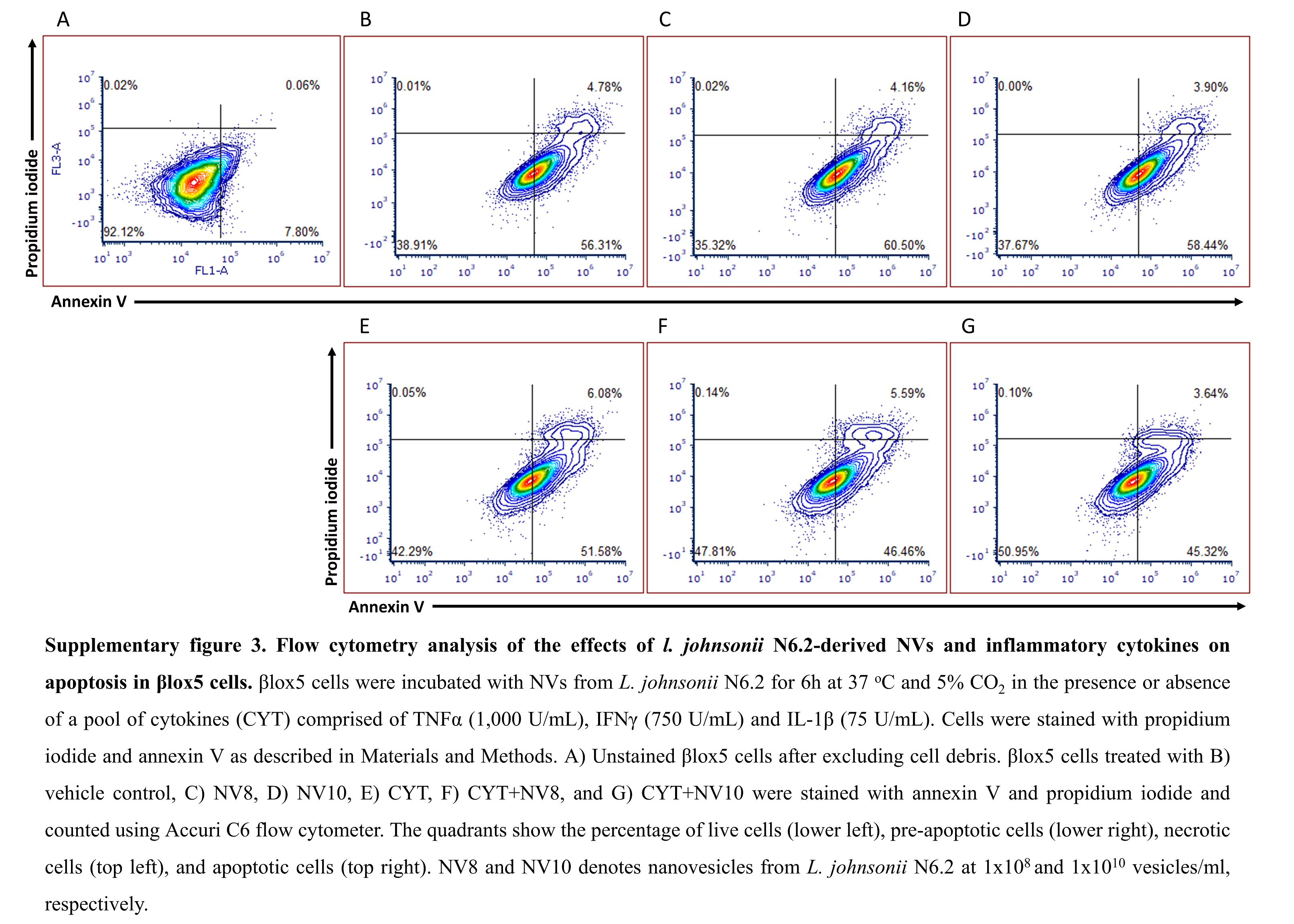

Supplement: Supplementary file 3 [file Image_3.tif]
